# Dissecting the Molecular Properties of Prokaryotic Flotillins

**DOI:** 10.1371/journal.pone.0116750

**Published:** 2015-01-30

**Authors:** Juri Niño Bach, Marc Bramkamp

**Affiliations:** Department of Biology I, Ludwig-Maximilians-University, Munich, Germany

## Abstract

Flotillins are universally conserved proteins that are present in all kingdoms of life. Recently it was demonstrated that the *B. subtilis* flotillin YuaG (FloT) has a direct influence on membrane domain formation by orchestrating lipid domains. Thereby it allocates a proper environment for diverse cellular machineries. YuaG creates platforms for signal transduction, processes crucial for biofilm formation, sporulation, competence, secretion, and others. Even though, flotillins are an emerging topic of research in the field of microbiology little is known about the molecular architecture of prokaryotic flotillins. All flotillins share common structural elements and are tethered to the membrane N’- terminally, followed by a so called PHB domain and a flotillin domain. We show here that prokaryotic flotillins are, similarly to eukaryotic flotillins, tethered to the membrane via a hairpin loop. Further it is demonstrated by sedimentation assays that *B. subtilis* flotillins do not bind to the membrane via their PHB domain contrary to eukaryotic flotillins. Size exclusion chromatography experiments, blue native PAGE and cross linking experiments revealed that *B. subtilis* YuaG can oligomerize into large clusters via the PHB domain. This illustrates an important difference in the setup of prokaryotic flotillins compared to the organization of eukaryotic flotillins.

## Introduction

Membrane microdomains, also termed lipid rafts, are conserved structures that occur in all kingdoms of life. These microdomains are enriched in distinct lipids and proteins [1]. A class of proteins that can routinely be found in lipid rafts are flotillins [2]. Flotillins are conserved proteins from bacteria to man. Generally, it is assumed that flotillins may act as scaffolding proteins for various biological processes like signalling, endo—exocytosis, transport, protein translocation and cell division [3–5]. All flotillins share common structural elements. They are attached to the membrane by a trans-membrane helix or a hairpin loop [6–8]. Eukaryotic flotillins are often tethered to the membrane by posttranslational modifications like palmitylation or myristylation. These modifications occur at the PHB (prohibitin) domain that is also often referred as SPFH (stomatin/prohibitin/flotillin/HflKC) domain [9, 10]. The PHB domain is located towards the N-terminus of the protein and was identified by various independent sequence homology blasts [9, 11–15]. Next to the PHB domain flotillins exhibit EA rich coiled–coil regions, also named flotillin domain [16]. The closest homologue to eukaryotic flotillins in bacteria is the *Bacillus subtilis* protein YuaG (FloT). It is 35.4% identical (67.1% homology) to *Mus musculus* Flotillin2 [17]. Notably, *B. subtilis* has a second flotillin homologue, YqfA (FloA) [18]. *B. subtilis* flotillins are highly dynamic and involved in sporulation, biofilm formation and cell wall synthesis [17–20]. Using mass spectrometry analysis it could be shown that eukaryotic lipid rafts are highly enriched in cholesterol [21]. Drugs that sequester or remove cholesterol result in disruption of lipid raft formation [22–24]. However, the *B. subtilis* membrane does not contain sterols. Previous reports indicated that YuaG domain formation is dependent on the protein YisP that was thought to be a squalene synthase [18], though recent experiments proofed that YisP is a phosphatase catalysing the dephosphorylation of farnesyl diphosphate to farnesol [25]. Hence, it is assumed that either farnesol or a still unknown lipid component might fulfil the role of cholesterol in *B. subtilis* membranes.

Flotillins were found to be involved in diverse cellular processes, but the molecular function of flotillins remained largely unknown [26]. Since flotillins are considered to be lipid raft marker proteins it is speculated that they might act as scaffolding proteins for membrane microdomains [27–29]. Recently, we could demonstrate by utilization of the anisotropic dye Laurdan that in *B. subtilis* distinct liquid ordered membrane domains exist [20]. Further, wild type *B. subtilis* membrane organization was compared with flotillin null mutant strains. This revealed that liquid ordered domains coalesce in the absence of flotillins. These results indicate that flotillins have a direct influence on membrane organization. Flotillins might prevent accumulation of certain classes of lipids or rather their fatty acid chains. Disruption of these microdomains lead to coalescence of liquid ordered regions and thereby impair correct functionality of diverse cellular machineries as exemplary shown for the Sec machinery and cell wall synthetic machinery [19, 20]. Flotillins seem to be sufficient to orchestrate lateral segregation of lipids and thereby provide the correct lipid environment for these cellular machineries [20]. Prevention of coalescence of liquid ordered regions might simply be facilitated by protein—protein and protein—lipid / fatty acid interactions [10]. Hence, YuaG should have specific domains for lipid / fatty acid specificity and protein—protein interaction. Strikingly, flotillins create large oligomeric structures in mega Dalton size [28, 30]. Likely, the formation of large complexes is also crucial to scaffold membrane domains. Importantly, it could be shown that some eukaryotic flotillins oligomerize via their flotillin domain [13]. However, nothing is known about the oligomerization site of prokaryotic flotillins. Here we show by cross-linking experiments, size exclusion chromatography and blue native PAGE that the PHB domain of YuaG is contrary to eukaryotic flotillins sufficient to oligomerize, but is likely not involved in lipid-binding. It is important to understand how prokaryotic flotillins bind to the membrane since the lipid binding domain might also be involved in orchestrating lipid domains. The topology of YuaG was analysed by SNAP-tag labelling experiments. YuaG is tethered to the membrane by a hairpin loop, similar to the topology of most eukaryotic flotillins [8]. This is an important hint that the hairpin loop is a conserved feature of flotillins and indicates a direct function of this structure on the functionality of prokaryotic flotillins.

## Material and Methods

### Strain construction

The strains, plasmids and oligonucleotides used in this study are listed in Tables 1–3, respectively.

**Table 1:** Oligonucleotides used in this study.

**Oligonucleotide**	**Sequence**	**Restriction site**
YuaG-PHB-f	TTACCCGGGGCGGCCGAACAATTTTTAGGGAAATCAAAAGACGACCGTG	SmaI
YuaG-PHB-r	GCTGTCGACTTCTATTTGTTTTTG	SalI
PHB-pSG1154-f	ATCGGTACCGCGGCCGAACAATTTTTAGGGAAATCAAAAGACGACCGTG	KpnI
PHB-pSG1154-r	GGCAAGCTTTTCTATTTGTTTTTG	HindIII

**Table 2:** Plasmids used in this study.

**Plasmid**	**Characteristics**	**Reference/Source**
pET52b	*bla lacI PT7 strepII his*	*Novagen*
pSG1154	*amyE3’ spc Pxyl-gfpmut1 amyE5’*	Lewis & Marston, 1999 [52]
PHB-pSG1154	*amyE3’ spc Pxyl-PHB-gfpmut1 amyE5’*	this work
pET52b_*yuaG*	*bla lacI PT7-strepII-yuaG-10xhis*	Bach & Bramkamp, 2013 [20]
pET52b-yuaG-PHB	*bla lacI PT7-strepII-yuaG-PHB-10xhis*	this work

**Table 3:** *E. coli* and *B. subtilis* strains used in this study.

** **	**Strains**	**Genotype**	**Reference/Source**
***E. coli***			
	DH5a	F- Φ80lacZM15 (lacZYA-argF)U169 recA1 endA1 hsdR17(rk^-^,mk^+^) phoA supE44 thi-1 gyrA96 relA1 λ^-^	Invitrogen
	BL21(DE3)	F− *ompT gal dcm lon hsdSB*(r−*B* m−*B*) _ (DE3 [*lacI lacUV5*-T7gene 1 *ind1 sam7 nin5*])	Studier & Moffatt, 1986 [53]
***B. subtilis***			
	DB002	*trpC2 amyE::(spc Pxyl-SNAP-yuaG)*	Donovan & Bramkamp, 2009 [17]
	IW001	*trpC2 amyE::(spc Pxyl-SNAP)*	Donovan & Bramkamp, 2009 [17]
	BB011	*trpC2 amyE::(spc Pxyl-PHB-GFP)*	PHB-pSG1154 —> 168

Plasmid amplification was carried out in *E. coli* DH5α. The amplified DNA was digested by restriction enzymes (NEB) and all constructed plasmids were verified by DNA sequencing (GATC) or by in house sequencing service. In the case of pet52b-*yuag*-PHB the sequence was amplified using the primers YuaG-PHB-f and YuaG-PHB-r. The construct was cloned into pET52b (Novagen) and subsequently transformed into *E.coli* BL21(DE3). The coding region for the PHB domain was amplified using the primers PHB-pSG1154-r and PHB-pSG1154-f, cloned into pSG1154 resulting in PHB-pSG1154 that was transformed into *Bacillus subtilis*.

### *B. subtilis* growth conditions

*B. subtilis* cells were grown in CH medium [31] or LB at 37°C. Strains were always freshly inoculated to an OD_600_ of 0.05 from an overnight culture and grown to stationary phase before adding xylose to a final concentration of 0.01–0.1%. Cells were further incubated at 37°C shaking before analysis.

### Labelling of SNAP-YuaG and free SNAP for microscopy

Labelling of SNAP-YuaG or free SNAP for microscopy was performed as described before [17]. Briefly, for labelling with the cell permeable SNAP-Cell TMR-Star (NEB) (excitation maxima: 554 nm; emission maxima: 580 nm) or the cell impermeable SNAP-Surface 488 (NEB) (excitation maxima: 506 nm; emission maxima: 526 nm) 1 µl of the stock solution was added to 400 µl culture and incubated for 30 minutes at 37°C. Cells were washed twice with fresh CH media,incubated for 15 minutes at 37°C and analysed microscopically.

Microscopy was performed as described in Bach et al. 2014 [32]. Images were taken on Zeiss AxioImager M1 equipped with a Zeiss AxioCam HRm camera. An EC Plan-Neofluar 100x/1.30 Oil Ph3 objective was used. Digital images were acquired with the AxioVision (Zeiss) software and analysed using the Axiovision 4.6 software (Zeiss). Final image preparation was done in Adobe Photoshop CS2 (Adobe Systems Incorporated).

### ProteinaseK digestion and in gel-fluorescence assay

*B. subtilis* was grown as described above. For each sample 400 µl cells were pelleted (5,000x*g*, 1 min, 37°C) and resuspended in 20 µl MSMNB (500 mM sucrose, 20 mM MgCl_2_, 20 mM malic acid, 13 g l^-1^ nutrient broth, pH 7.5) and 15 nm SNAP-CellCellCell TMR-Star (NEB) final concentration was added. Lysozyme was added to a final concentration of 2 mg ml^-1^ from a 10 mg ml^-1^ stock solved in MSMNB, cells were incubated at 37°C without shaking and protoplastation was followed microscopically. ProteinaseK was added to a final concentration of 0.1 mg ml^-1^ from a 1 mg ml^-1^ stock solved in MSMNB. ProteinaseK digestion was stopped by addition of 10 mM phenylmethanesulfonylfluoride (PMSF) final concentration. Samples were mixed with 10 µl 4x SDS-PAGE loading dye, cooked for 20 minutes at 95°C and analysed via SDS-PAGE. In gel-fluorescence was analysed using a Typhoon Trio scanner (Amersham Biosciences) using a 532 nm laser and a 580 nm emission filter. Quantification of fluorescence was performed using ImageJ [33].

### Preparation of cell lysates, cytoplasm and membranes

*E. coli* cells were grown in Luria-Bertani (LB) medium containing carbenicillin 50 µg ml^-1^. For protein production the T7 RNA polymerase based pET system (Novagen) in *E. coli* BL21(DE3) was used. Freshly transformed cells were grown overnight and diluted into LB medium to OD_600_ = 0.1. Cells were grown at 30°C at 170 rpm. At OD600 = 0.8 cells were induced with 10 µM IPTG. Expression was performed for 2 h.

*B. subtilis* cells were grown in LB medium to stationary phase at 37°C shaking. *B. subtilis* and *E. coli* cells were harvested at 5,000x*g* for 10 minutes at 4°C. Cell pellets were washed twice in buffer A (50 mM TrisHCl pH 7.5; 150 mM NaCl; 5 mM MgCl2) at 4°C. Cells were processed directly or flash frozen and stored at -80°C.

Cells were resuspended in 5–8 volumes in buffer A and DNase I was added in appropriate amounts. Cells were disrupted in a French press homogenizer at 125 MPa for 3–5 passes. The suspension was centrifuged for 15 minutes at 4°C and 12,000 *g* and the cell debris was removed. Membranes were pelleted by ultracentrifugation > 200,000x*g* for 120 minutes at 4°C. For preparation of cytoplasm the supernatant was used, for membrane isolation the membranes were resuspended in 5 ml buffer A and processed directly or flash frozen.

### Polyacrylamide gel electrophoresis

SDS-PAGE was performed according to Laemmli [34]. Blue native PAGE was performed according to Wittig et al. [35].

### Purification of YuaG-PHB domain

The cleared lysate was loaded to a HisTrap FF crude (1 ml) column (GE Healthcare). Column chromatography was performed at RT using an ÄKTA Explorer (GE Healthcare). Protein was detected by absorbance at 280 nm. The column was washed with 10 column volumes (CV) buffer A. Subsequently, the column was washed stepwise with 5 CV buffer A containing 25 or respectively 50 mM imidazole. Protein was eluted with buffer A containing 250 mM imidazole. Purification was controlled by SDS-PAGE and α-His immunoblot. For size exclusion chromatography (SEC) the protein was loaded on a Superose 6 10/300 GL column (GE Healthcare) using a 500 µl loop. SEC was performed in buffer A. Calibration of the column was performed with the Gel Filtration Standard (BIO-RAD, #151–1901) using a linear fit of logarithmic mass / kDa against the elution constant K_av_ = (V_e_-V_0_)/(V_t_-V_0_) with V_e_ = elution volume of protein; V_t_ = total volume; V_0_ Elution volume of aggregate. SEC was controlled by SDS-PAGE and α-His immunoblot.

### CD Spectroscopy of PHB domain

Freshly affinity purified PHB domain was loaded on a Superdex200 GL column (GE Healthcare). SEC was performed in buffer B (40 mM NaF, 20 mM sodium phosphate buffer pH 7.4) and controlled by SDS-PAGE and α-His immunoblot. Protein concentration was determined using BCA assay (Pierce) CD spectroscopy was performed using 0.1 mg ml -1 PHB domain diluted in buffer B on a Jasco J-810 spectropolarimeter in a 1 mm quartz cuvette from 178–260 nm and data corresponded to an average of 20 repeated scans. CD spectroscopy using pure buffer B was performed as a control.

### Cross-linking of the PHB domain

The PHB domain was cross-linked using 0.1% formaldehyde final concentration for 15 minutes at 30°C. Cross-linking was stopped by the addition of 100 mM glycine final concentration.

### Lipid-binding experiments

*E. coli* polar lipid extract (Avanti Polar Lipids) was dried in a rotational evaporator and lyophilized overnight. Lipids were resuspended in buffer A at 20 mg/ml and stored at -80°C. *B. subtilis* lipids / membranes were isolated as described above. For liposome preparation, *E. coli* lipids were diluted to 5 mg ml^-1^ in buffer A. Either *E. coli* lipids or isolated *B. subtilis* membranes were extruded 20 times through a 400 nm pore size membrane (Millipore). 1 ml of liposomes were incubated for 1h at RT with 100 µl of PHB domain solution (0.8 mg ml^-1^) rolling with 0.5 revolution per second. Protein concentration was determined using BCA assay (Pierce). As a control the PHB domain was incubated with buffer only and used for all steps mentioned below. The mixture was centrifuged at 200,000x*g* for 20 minutes at 4°C. The pellet was resuspended in an equal volume and centrifuged again. Finally the pellet was again resuspended in an equal volume. All samples were analysed by SDS-PAGE. Sedimentation of DynA was performed identical to PHB domain. DynA was purified according to Bürmann et al, 2011 [36].

## Results and Discussion

**Figure 1:**
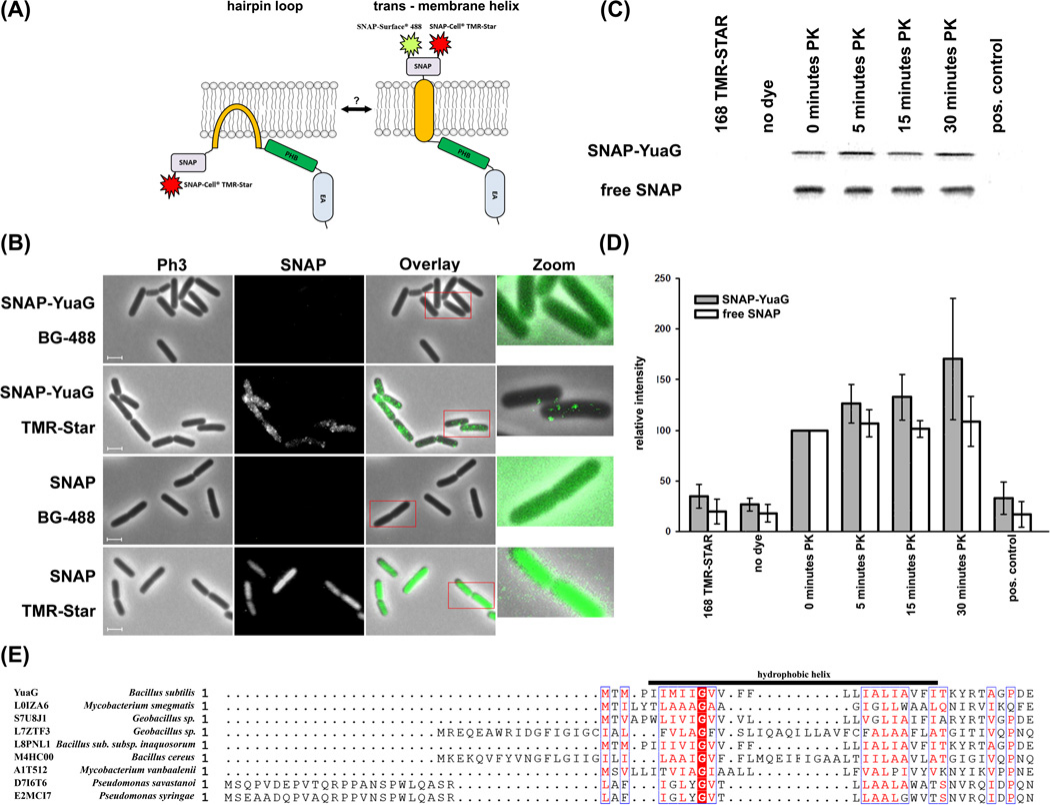
Topology of YuaG. (A) A cartoon of the two putative topologies of SNAP-YuaG is shown. YuaG has either a hairpin loop tethering it to the membrane or a trans-membrane helix as predicted by bioinformatics tools. SNAP dye TMR-Star is membrane permeable and hence sufficient to label in- and outside the cell, labelling with SNAP dye BG-488 (SNAP-surface 488), which is membrane impermeable, is only possible with an extracellular SNAP tag. (B) Cells expressing SNAP-YuaG or free SNAP were labelled with the cell impermeable SNAP dye BG-488 and the cell permeable SNAP dye TMR-Star. SNAP dyes are all false coloured in green. Zoomed in regions are indicated with a red frame. Scale bar 2 µm. (C) A representative Proteinase K (PK) sensitivity assay is shown for protoplasted *B. subtilis* cells. Control experiments using wild type cells incubated with TMR-Star and unlabeled cells expressing SNAP-YuaG confirmed that TMR-Star does not unspecifically label *B. subtilis* proteins. As a positive control protoplasts were resuspended in water instead of MSMNB and TritonX-100 was added to a final concentration of 1%. (D) The in gel-fluorescence of the PK assay was quantified. Fluorescence of 0 minutes PK was always defined as 100%. Standard deviation is shown; n = 4. (E) Alignment of the hydrophobic helix of *B. subtilis* YuaG with different flotillins from various bacteria. Note the conserved glycine residue, highlighted in red. The alignment was performed using Kalign with default settings [43].

YuaG (FloT) is a 55.8 kDa protein with a predicted hydrophobic membrane anchor (amino acids (AA) 5–24). Eukaryotic flotillins are tethered to the membrane either by a trans-membrane helix or by a hairpin loop with additional lipid modification within the PHB domain [6–8]. To discriminate between these topologies we used a YuaG construct with an N’-terminally fused SNAP tag (Fig. 1A). This construct has already been shown to be functional *in vivo* with respect to sporulation in *B. subtilis* [17]. We incubated cells expressing SNAP-YuaG with the membrane impermeable SNAP dye BG-488 (NEB) and the membrane permeable dye SNAP-CellCell TMR-Star (NEB). If YuaG has a trans–membrane helix with an outward facing N-terminus, it should be possible to label the protein with both dyes. Contrary, if the hydrophobic helix adopts a hairpin structure, it should not be possible to label SNAP-YuaG with BG-488. Notably, a similar approach was used by Schurek et al. 2014 who showed by FLAG-tag labelling experiments with membrane permeable and impermeable substrates that podocin is tethered to the membrane by a hairpin loop [37]. Indeed, our labelling results clearly demonstrate that SNAP-YuaG can be labelled with SNAP-CellCell TMR-Star but not with BG-488 (Fig. 1B). Admittedly, a significant amount of SNAP-YuaG seems to be degraded as detected by SDS-PAGE and in gel-fluorescence leading to diffuse cytosolic background staining of varying degree in each cell (S1 Fig.) [17]. However, close-up images of single cells proof the existence of SNAP-YuaG foci (Fig. 1B). Additional controls demonstrate that the soluble SNAP protein, expressed in the cytoplasm of *B. subtilis,* can only be labelled with the membrane permeable dye SNAP-CellCell TMR-Star and not with BG-488 (Fig. 1B), showing the functionality of the assay. These results indicate that the N’-terminus of YuaG is cytoplasmatic, thereby suggesting that *B. subtilis* flotillin is tethered to the membrane by a hairpin loop. To additionally proof the topology of YuaG we performed protease sensitivity studies. Therefore, we protoplasted cells expressing SNAP-YuaG using lysozyme and treated protoplasts with Proteinase K (PK). Quality of the protoplasts was checked by phase contrast microscopy. We used in gel fluorescence of TMR-Star labelled cells to visualize SNAP-YuaG. Full length SNAP-YuaG was readily detected after labelling and control experiments confirmed that TMR-Star does not label *B. subtilis* proteins unspecific. After addition of PK the intensity of the band corresponding to full length SNAP-YuaG remains constant over a time of 30 minutes. The full length SNAP-YuaG is, however, degraded when protoplasts were lysed by the addition 1% TritonX-100 final concentration (Fig. 1C, positive control; for experimental details see material and methods). The whole gel can be found in the S1 Fig. Similar results were obtained for labelling of free SNAP protein. To get a more quantitative analysis we plotted intensities of four individual experiments (Fig. 1D). The amount of soluble SNAP remains constant before addition of Triton X-100. The intensities of SNAP-YuaG even increase slightly, which may be a consequence of continuing expression. The results of the protease sensitivity assay indicate that YuaG is tethered to the membrane by a hairpin loop rather than by a trans-membrane helix. A model with two transmembrane domains is unlikely, given the fact that hydrophobicity plots indicate only one hydrophobic domain that could span the membrane twice. The predicted hydrophobic helix is too short (AA) 5–24) to cross the membrane. The topology of a hairpin loop in eukaryotic flotillins is often achieved by a proline residue that acts as a helix breaker inside the hydrophobic helix [38]. Notably, YuaG does not contain any proline residue inside the hydrophobic region (AA5–24). However, an alignment of YuaG with various prokaryotic flotillins revealed a conserved glycine residue in prokaryotic flotillins (Fig. 1E). Importantly, glycine can also act as a helix breaker in alpha helices [39]. Hence, we speculate that this residue is critical for corrected assembly of the amphiphatic helix or rather the hairpin loop. However, it should be noted that not all prokaryotic flotillin homologs reveal a hydrophobic helix with a central glycine residue, suggesting that maybe different membrane anchor mechanisms exist. We speculate that hairpin loops in flotillins are likely crucial to fulfil functions related to membrane organization in the inner leaflet of the membrane. Examples are the hairpin loops in podocin, Mec-2 and stomatin which are required to localize the protein in detergent resistant membranes [37, 38].

**Figure 2:**
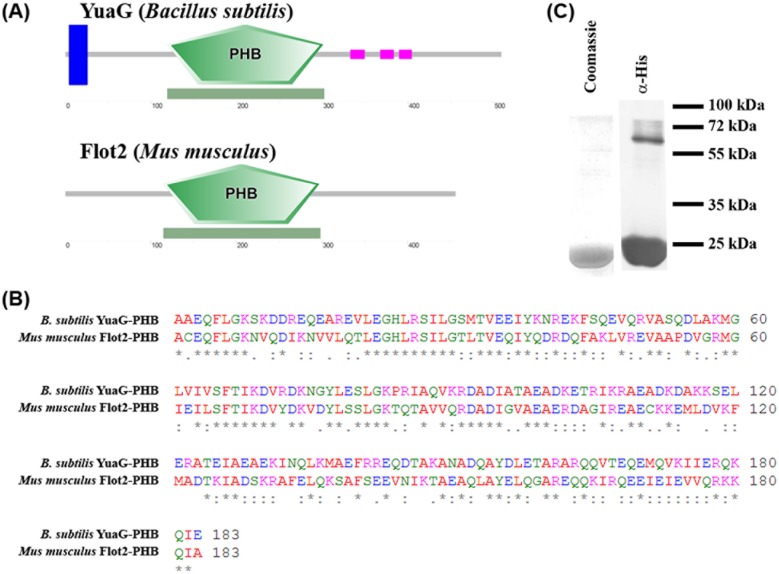
Analysis of the PHB domain of YuaG. (A) The PHB domain of *B. subtilis* YuaG is a close homologue of the PHB domain of *Mus musculus* flotillin2. A cartoon (blue trans-membrane helix, green PHB domain, purple coiled-coiled regions) of the predicted topology [40, 41] of *B. subtilis* YuaG and *M. musculus* flotillin2 is shown. (B) A ClustalW alignment [42–44] was performed with *B. subtilis* YuaG PHB domain and *M. musculus* Flot2 PHB domain. The alignment revealed that the PHB domains of both flotillins are 43.8% identical and share a similarity of 79.1%. (C) The PHB of domain of YuaG was purified by metal affinity chromatography and analysed by SDS-PAGE and subsequent immunoblotting.

**Figure 3:**
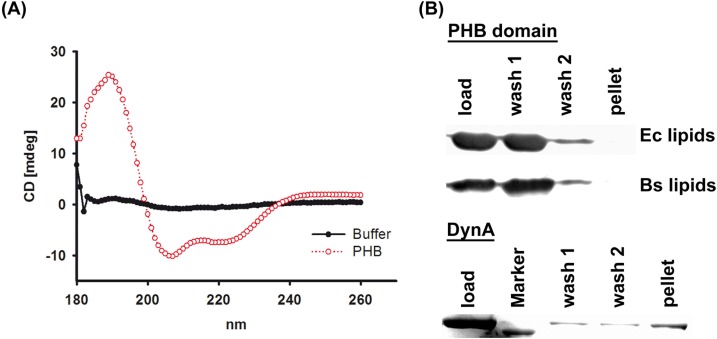
Lipid binding of the PHB domain. (A) CD-spectroscopy analysis of the purified PHB domain (red line) and a buffer control (black line). (B) The purified PHB domain and purified DynA (positive control) were incubated with liposomes. After 1h liposomes were sedimented, washed and finally analysed via SDS-PAGE and α-His Immunoblot (PHB domain) or Coomassie blue staining (DynA). The *B. subtilis* PHB domain does not bind to *B. subtilis* and *E. coli* liposomes and cannot be detected in the pellet fraction. DynA binds to liposomes and co-sediments with the membrane.

A typical characteristic of flotillins is that they contain a PHB domain. The PHB domain of YuaG is 43.8% identical and 79.1% similar to the PHB domain of Flot2 from *M. musculus* and both proteins show a similar predicted topology (Fig. 2A) [40, 41]. A ClustalW alignment of both PHB sequences is shown in Fig. 2B [42–44]. To test if the PHB domain of *B. subtilis* YuaG is sufficient to bind to membranes as known for eukaryotic flotillins [13] we heterologously expressed the PHB domain of *B. subtilis* YuaG in *E. coli* and purified it via nickel affinity chromatography (Fig. 2C). The purity of the purified PHB domain was controlled by SDS-PAGE with subsequent Coomassie staining and α-His-immunoblot (Fig. 2C). Proper folding of the PHB domain was controlled by CD-spectroscopy (Fig. 3A; for experimental details see material and methods). The CD spectrum reveals two distinct minima around 207 and 219 nm. These minimal are indicative of alpha-helices and, hence, a sign for protein folding. To probe the PHB domain for lipid binding the protein was incubated with liposomes composed of *E. coli* or *B. subtilis* lipids, pelleted by ultra-centrifugation, washed and finally analysed via α-His immunoblot (see material & methods). Notably, YuaG does not contain any cysteine residues that could be modified and thereby contribute to lipid binding. The PHB domain was only detected in the supernatant and, hence, did not bind to liposomes (Fig. 3B). This was true for liposomes made from *E. coli* lipids and *B. subtilis* lipids. As a positive control experiment we used the bacterial dynamin DynA, which has been shown to bind membranes *in vivo* and *in vitro* [36, 45]. We also fused the PHB domain to GFP and analysed the fusion construct after expression in *B. subtilis* cells microscopically. Only a weak signal above background was detected, indicating that this fusion construct is degraded rapidly (S2 Fig.). However, the entire PHB-GFP signal was localized diffuse within the cytoplasm, ruling out that the fusion protein is recruited to the membrane (S2 Fig.).

**Figure 4:**
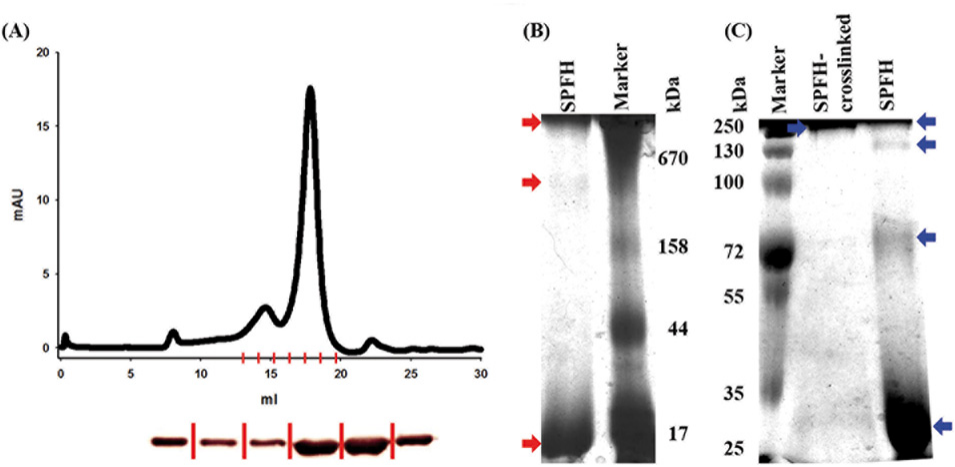
Oligomerization of the PHB domain. (A) Size exclusion chromatography was performed with the PHB domain on a Superose6 column (GE Healthcare) and two elution peaks were detected at 14.6 and 17.5 ml, corresponding to a size of 313 kDa and 42.8 kDa respectively. (B) Blue native PAGE was performed with the PHB domain, three bands marked with red arrows could be detected corresponding to a monomer, a 14mer (313 kDa) and an oligomer larger than 670 kDa. (C) A colloidal Coomassie blue stained SDS—PAGE gel is shown. The PHB domain was further probed by cross-linking and applying the sample to SDS-PAGE. For cross-linked PHB only a band larger than 250 kDa is detected, for none cross-linked PHB domain bands corresponding to a monomer, a tetramer, an octamer and an oligomer larger than 250 kDa are be detected (indicated by blue arrows). The SEC column was calibrated using standard proteins (see S2 Fig.).

Flotillins are characterized by the existence of a conserved PHB domain and an adjacent EA rich coiled—coil regions. Eukaryotic flotillins oligomerize via their EA rich coiled—coil regions [13] and are tethered to the membrane via their PHB domain. We wanted to investigate the role of the PHB domain in *B. subtilis* YuaG, in order to understand the molecular function of this domain. To test if the *B. subtilis* PHB domain is, contrary to eukaryotic flotillins, involved in flotillin oligomerization we analysed the oligomeric state of the isolated PHB domain. Immunoblot analysis of the isolated PHB domain revealed that even under denaturing SDP-PAGE conditions an oligomeric PHB complex can be detected (Fig. 2C). Next we analysed the oligomerization state of the PHB domain via size exclusion chromatography (SEC). Two peaks at 14.6 ml and 17.5 ml could be detected (Fig. 4A). These peaks correspond to a size of 313 kDa (higher oligomer corresponding to a theoretic 14.8mer) and 42.8 kDa (monomer/dimer), respectively. The elution profile of a protein standard with known molecular weights is shown in S3 Fig. Immunoblot analysis of the SEC fractions revealed that all peaks contain the PHB protein (Fig. 4A). To additionally test the oligomerization properties of the PHB domain, the isolated protein was analysed via blue native polyacrylamide gel electrophoresis (BN-PAGE). The BN-PAGE revealed that three distinct oligomerization states can be found for the PHB domain. The lowest band was detected at a size 21 kDa, in good accordance with a monomeric PHB domain (Fig. 4B; lower red arrow), suggesting that the SEC peak at 17.5 ml might indeed represent monomeric protein (note that the Superose 6 column does not separate well enough in this range). The BN-PAGE also reveals bands corresponding to a ~14-mer (Fig. 3B; middle red arrow) and an oligomer > 670 kDa (Fig. 4B; top red arrow). It remains elusive if the oligomer > 670 kDa represents a true oligomerization state of the PHB domain, since the only corresponding peak that could be found using size exclusion chromatography is close to the void volume (8 ml). Additionally, SDS-PAGE was performed with formaldehyde cross-linked PHB domain (see material and methods) and the native PHB domain. Only one band can be found for cross-linked PHB domain analysed via SDS-PAGE and subsequent colloidal Coomassie blue staining [46]. This band runs higher than 250 kDa (Fig. 4C). For the control without cross-linking, four fractions partially resistant to SDS denaturation with sizes corresponding to a monomer, a tetramer, an octamer and a higher oligomer could be detected (Fig. 4C).

It is commonly accepted that flotillins fulfil similar functions in all organisms. Owing to their conserved function, flotillins also share a common topology [16]. Most eukaryotic flotillins are tethered to the membrane via a hairpin loop and via palmitoylation or myristylation [9, 10]. Though, a transmembrane helix is predicted for *B. subtilis* flotillin YuaG leading to an outward facing N-terminus. We could show here, contrary to the predicted topology, that YuaG is tethered to the membrane by a hairpin loop and that the N-terminus of YuaG is facing the cytoplasm. A putative candidate that might trigger bending of the hydrophobic helix and thereby facilitate the hairpin structure is a glycine residue (Gly10). Although, it has to be mentioned here that we cannot fully exclude that N’-terminal fusion of SNAP-tag to YuaG might somehow alter protein translocation mechanisms that might process YuaG, the functional complementation of SNAP-YuaG [17] and the fact that correct membrane insertion is crucial for function of PHB domain proteins [37, 38], it is unlikely to assume an artificial membrane insertion of SNAP-YuaG. Interestingly, for some eukaryotic PHB domain proteins it was shown that the protein may exist in different topologies. Exemplary, stomatin can exist in at least two distinct conformations harbouring either a hairpin loop or a trans-membrane helix, although the molecular and biological reason for this phenomena remains elusive [38]. The topology of membrane helices may also be dependent on the correct lipid environment. *E. coli* LacY exists in different topologies dependent on the amount of phosphatidylethanolamine present in the membrane system [47]. Hence, it cannot be fully excluded that further factors exist which might alter YuaG topology. Further, we could show by various molecular approaches that the molecular architecture of prokaryotic flotillins is similar to the architecture of eukaryotic flotillins. We could demonstrate here, that the PHB domain of *B. subtilis* YuaG does not bind to lipids. It is often speculated that the eukaryotic PHB domain containing proteins act as a scaffolding elements for membrane rafts [2, 48]. The PHB domain is crucial to target eukaryotic flotillins to specific membrane domains [11]. Since the PHB domain of YuaG does not bind to membranes and the full length YuaG also lacks any cysteine that could be palmitoylated or myristoylated it is unlikely that YuaG PHB domain is involved in membrane microdomain binding or folding specificity. Oligomerization and protein—protein interaction are crucial elements for flotillins to orchestrate lipid domains [20, 26, 28, 30, 49]. In eukaryotic flotillins this is mediated via the flotillin domain [13]. In contrast to that we could show here that the PHB domain of YuaG is sufficient for oligomerization. A similar behaviour could be shown for flotillins of the archaeum *Pyrococcus horikoshii* [50]. Though, it remains elusive if further elements like post translational modifications are required to facilitate oligomerization in all classes of flotillins. Nonetheless, oligomerization into huge assemblies probably in MDa size is a common future of all flotillins [2, 20, 28, 50, 51]. The same molecular setup of prokaryotic and eukaryotic flotillins with slightly different molecular, but similar cellular functions indicate that flotillins are way more complex scaffolding elements than previously anticipated and fulfil critical function in pro- and eukaryotes.

## Supporting Information

S1 Fig Proteinase K sensitivity assay with protoplasted cells. SDS-PAGE and in gel-fluorescence of wild type cells incubated with TMR-Star, cells expressing SNAP-YuaG incubated without and with TMR-Star and Proteinase K (PK). Degraded SNAP-YuaG bands are visible. Note the complete degradation of SNAP-YuaG after addition of Triton-X 100 (positive control) to the cells.
(TIF)

S2 Fig The soluble PHB domain of YuaG does not bind to membranes *in vivo*. Cells expressing PHB-GFP labelled with FM4–64 are shown. Scale bar 2 µm. Note the cytoplasmic localization of the GFP fusion protein.
(TIF)

S3 Fig Calibration of gel-filtration column. Elution profile of the molecular mass standard used for size-exclusion calibration (BioRad; #151–1901). Peaks are labelled with *. Molecular masses are from left to right: 670 kDa, 158 kDa, 44 kDa, 17 kDa, 1.35 kDa.
(TIF)
