# Supplementary figures and images for: Dissecting the Molecular Properties of Prokaryotic Flotillins
Source: PLoS One. 2015 Jan 30;10(1):e0116750. doi: 10.1371/journal.pone.0116750 (PMC4312047; doi:10.1371/journal.pone.0116750)

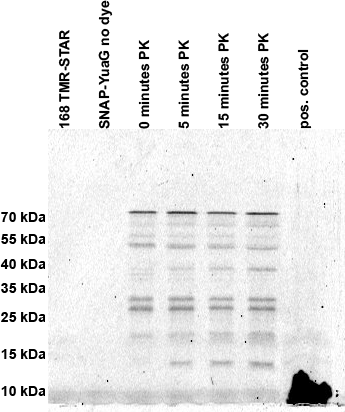

Supplement: S1 Fig — SDS-PAGE and in gel-fluorescence of wild type cells incubated with TMR-Star, cells expressing SNAP-YuaG incubated without and with TMR-Star and Proteinase K (PK). Degraded SNAP-YuaG bands are visible. Note the complete degradation of SNAP-YuaG after addition of Triton-X 100 (positive control) to the cells. (TIF) [file pone.0116750.s001.tif]

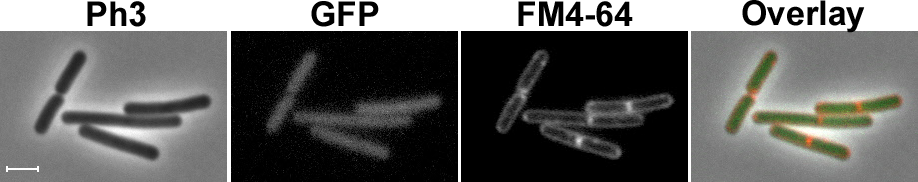

Supplement: S2 Fig — Cells expressing PHB-GFP labelled with FM4–64 are shown. Scale bar 2 µm. Note the cytoplasmic localization of the GFP fusion protein. (TIF) [file pone.0116750.s002.tif]

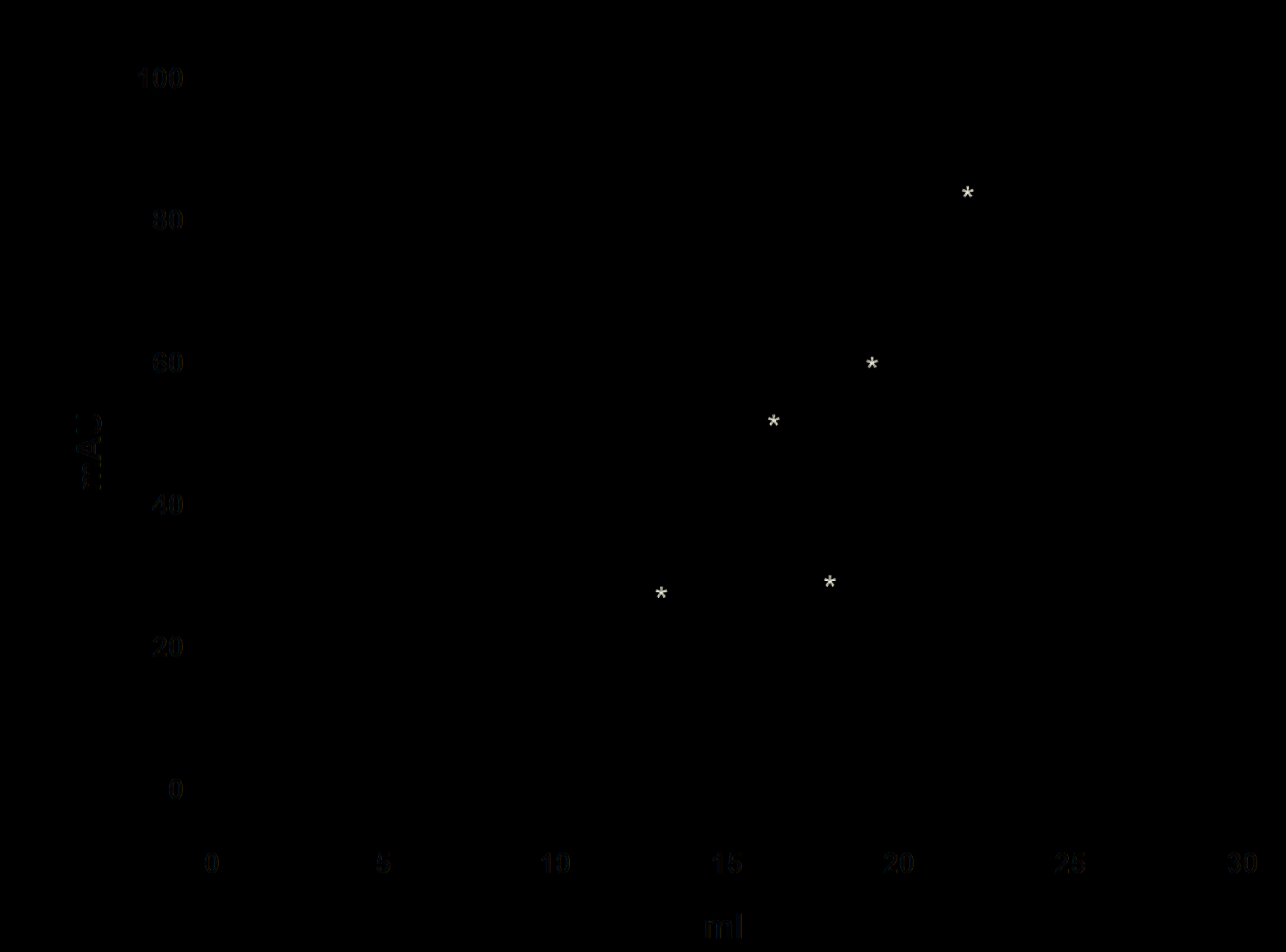

Supplement: S3 Fig — Elution profile of the molecular mass standard used for size-exclusion calibration (BioRad; #151–1901). Peaks are labelled with *. Molecular masses are from left to right: 670 kDa, 158 kDa, 44 kDa, 17 kDa, 1.35 kDa. (TIF) [file pone.0116750.s003.tif]
